# Performance of ^18^F-FDG PET/CT Radiomics for Predicting EGFR Mutation Status in Patients With Non-Small Cell Lung Cancer

**DOI:** 10.3389/fonc.2020.568857

**Published:** 2020-10-08

**Authors:** Min Zhang, Yiming Bao, Weiwei Rui, Chengfang Shangguan, Jiajun Liu, Jianwei Xu, Xiaozhu Lin, Miao Zhang, Xinyun Huang, Yilei Zhou, Qian Qu, Hongping Meng, Dahong Qian, Biao Li

**Affiliations:** ^1^Department of Nuclear Medicine, Ruijin Hospital, Shanghai Jiao Tong University School of Medicine, Shanghai, China; ^2^Institute of Medical Robotics, Shanghai Jiao Tong University, Shanghai, China; ^3^Department of Pathology, Ruijin Hospital, Shanghai Jiao Tong University School of Medicine, Shanghai, China; ^4^Department of Oncology, Rujin Hospital, Shanghai Jiao Tong University School of Medicine, Shanghai, China

**Keywords:** positron emission tomography/computed tomography, radiomics, lung cancer, epidermal growth factor receptor, ^18^F-fluorodeoxyglucose

## Abstract

**Objective:**

To assess the performance of pretreatment ^18^F-fluorodeoxyglucose positron emission tomography/computed tomography (^18^F-FDG PET/CT) radiomics features for predicting EGFR mutation status in patients with non-small cell lung cancer (NSCLC).

**Patients and Methods:**

We enrolled total 173 patients with histologically proven NSCLC who underwent preoperative ^18^F-FDG PET/CT. Tumor tissues of all patients were tested for EGFR mutation status. A PET/CT radiomics prediction model was established through multi-step feature selection. The predictive performances of radiomics model, clinical features and conventional PET-derived semi-quantitative parameters were compared using receiver operating curves (ROCs) analysis.

**Results:**

Four CT and two PET radiomics features were finally selected to build the PET/CT radiomics model. Compared with area under the ROC curve (AUC) equal to 0.664, 0.683 and 0.662 for clinical features, maximum standardized uptake values (SUV_max_) and total lesion glycolysis (TLG), the PET/CT radiomics model showed better performance to discriminate between EGFR positive and negative mutations with the AUC of 0.769 and the accuracy of 67.06% after 10-fold cross-validation. The combined model, based on the PET/CT radiomics and clinical feature (gender) further improved the AUC to 0.827 and the accuracy to 75.29%. Only one PET radiomics feature demonstrated significant but low predictive ability (AUC = 0.661) for differentiating 19 Del from 21 L858R mutation subtypes.

**Conclusions:**

EGFR mutations status in patients with NSCLC could be well predicted by the combined model based on ^18^F-FDG PET/CT radiomics and clinical feature, providing an alternative useful method for the selection of targeted therapy.

## Introduction

Lung cancer is the leading cause of cancer-related death in the world (1). Non-small cell lung cancer (NSCLC) accounts for approximately 80% to 85% of all lung cancers (2). Epidermal growth factor receptor (EGFR) tyrosine kinase inhibitor (TKI) has become a first-line drug in the treatment of NSCLC. Because the efficacy of TKI therapy is closely related to EGFR mutation status, identification of mutation status before the administration of TKI is crucial in achieving the best curative effect. Furthermore, exon 19 deletion (19 del) and exon 21 L858R point mutation (21 L858R),the most common mutation subtypes of EGFR (3), demonstrate different clinical outcomes in patients with NSCLC after TKI treatment (4, 5). *Current molecular testing for identifying EGFR mutation status is mainly based on tumor tissue from biopsies and surgical resection* (6). However, focal tissue testing may sometimes be limited by invasive procedures or tissue samples that are not readily available (7), causing patients to lose potential opportunities for EGFR-TKI treatment.

Medical imaging can reflect tumor gene-driven phenotype (8). ^18^F-fluorodeoxyglucose (^18^F-FDG) PET/CT, as a noninvasive molecular imaging tool, has been widely used in the evaluation of glucose metabolic phenotype of tumor (6). Previous data has suggested that several genes associated with glucose metabolism, including GLUT1 (9), GPI, G6PD, PKM2, and GAPDH (10), are down-regulated in EGFR-mutated lung cancer. Therefore, numerous studies have explored the relationship between ^18^F-FDG PET/CT images and EGFR mutation status. Some studies suggested that there was significantly lower maximum standardized uptake values (SUV_max_) of NSCLCs with EGFR mutations than those with wild type (11–14), but other studies reported non-significant (15) or opposite results (16). These confusing findings may be related to intra-tumoral heterogeneity of EGFR mutation (17) that the PET-derived semi-quantitative parameters cannot well reflect.

Radiomics data obtained using mathematical algorithms can quantitatively describe the spatial relationship between voxels, and become an important tool to study tumor heterogeneity *in vivo* (18). To date, most studies using radiomics for the prediction of EGFR mutation status in NSCLC are based on chest CT images (19, 20), whereas few studies about the relationship between PET or PET/CT radiomics features and EGFR mutation status in lung cancer (21–23) are conducted.

In the present study, both PET and CT radiomics features that significantly discriminated EGFR mutation status were extracted and selected for establishing a robust predictive model. Then we compared the predictive performances of the radiomics model, clinical features, and conventional PET-derived semi-quantitative parameters. Moreover, we tried to investigate the possibility of PET/CT radiomics features for distinguishing the 19 del from the 21 L858R mutation which both are two main mutation subtypes of EGFR.

## Materials and Methods

### Subjects

A total of 173 patients (115 men, 58 women; mean [± SD] age 60.9 ± 10.9 years [range, 27–86 years]) with histologically proven NSCLC, who had undergone pre-treatment ^18^F-FDG PET/CT between January 2017 and March 2018, were included in this study. This retrospective study was approved by the Ethics Committee of Ruijin Hospital, Shanghai Jiao Tong University School of Medicine.

### ^18^F-FDG PET/CT Imaging

Patients were required to fast for at least 6 h before ^18^F-FDG PET/CT scan using GE Discovery VCT64 system, and their serum glucose levels were maintained to < 7.8 mmol/L. Whole-body imaging was performed approximately 60 min after the intravenous administration of 5.55 MBq of ^18^F-FDG per kilogram of body weight. Emission images were acquired for 3 min per bed position using 128 × 128 matrix size, 28 subsets, 2 iterations and full-width half-maximum post-filtering. CT images were acquired using 140 kV tube voltage, 220 mA tube current, and 3.75 mm section thickness. PET images were reconstructed based on an ordered-subset expectation maximization algorithm with photon attenuation correction from CT data.

### EGFR Mutation Status Analysis

Tissue samples from lung tumors were obtained through biopsy or surgical resection followed by 10% formalin fixation, paraffin embedding, and sectioning. After extracting DNA from sample sections, the nucleotide sequence encoding the kinase domain (exons 18-21) of EGFR was tested using an amplification refractory mutation system polymerase chain reaction (24) or target sequencing method based on polymerase chain reaction (25) using the X10 system (Illumina, San Diego, CA, USA).

### PET/CT Image Feature Extraction, Selection and Model Establishment

**Figure 1:**
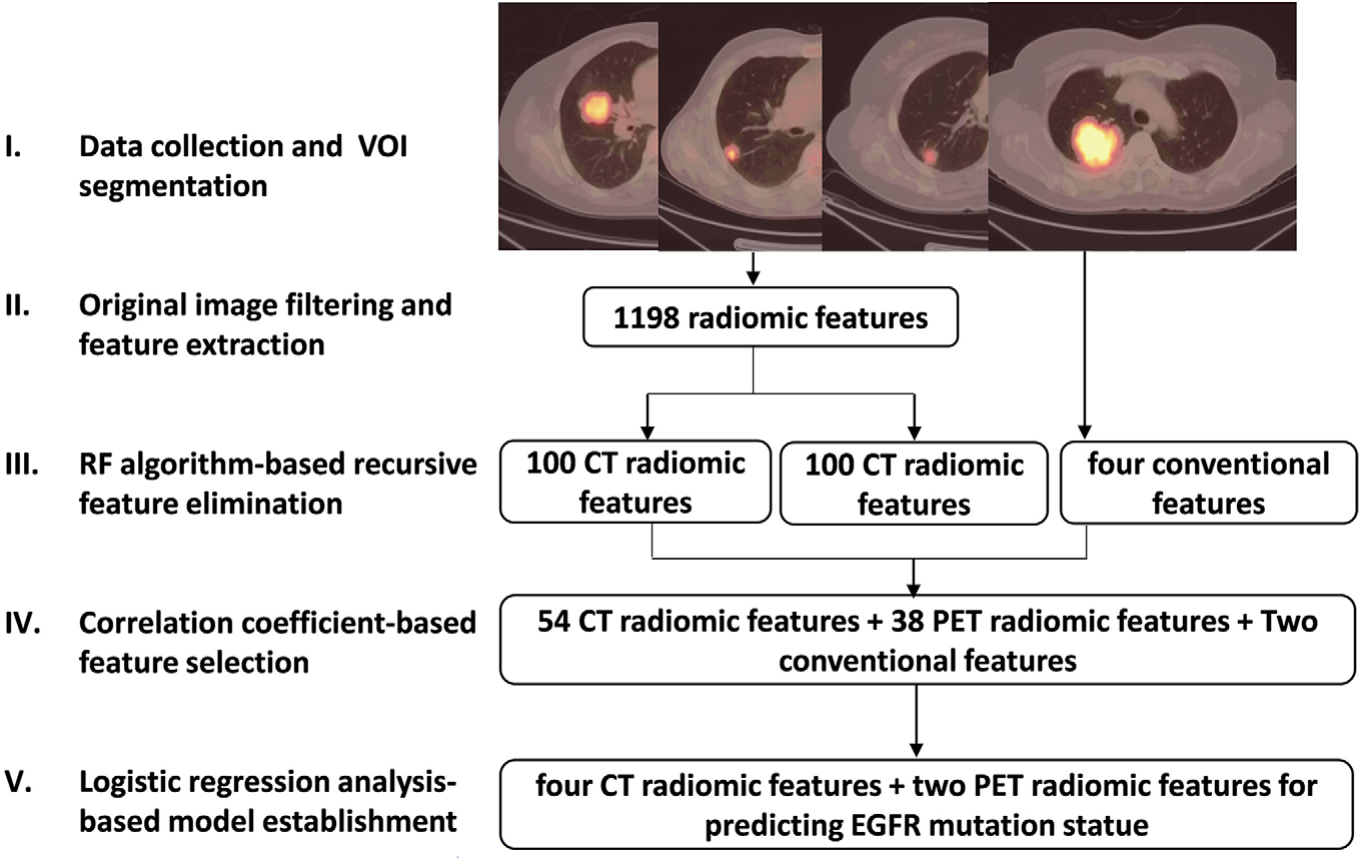
Schematic diagram of image feature extraction and selection steps.

All segmentation was performed by experienced nuclear medicine physicians blinded to the mutation data using an open-source ITK-SNAP software (version 3.6, https://www.itksnap.org) to manually outline the contour of the volume of interest on CT images, and automatically delineated on PET images using a fixed SUV_max_ threshold of 2.5 as previously reported (21). The extraction and selection of radiomics features were performed according to the following steps (**Figure 1**):

1. Before extraction of radiomics features, filters including Laplacian of Gaussian, wavelet, square, square root, logarithm and exponential (26) (**Supplemental Table 1**), were applied to the original PET/CT images to highlight image features for more efficient feature extraction.
2. Based on the original and filtered PET/CT images mentioned above, several types of well-designed image features were calculated using pyradiomics python package (27). These features are designed in compliance with the Image Biomarker Standardization Initiative (28) including First-order statistics, Shape, Gray Level Co-occurrence Matrix (GLCM), Gray Level Size Zone Matrix (GLSZM), Gray Level Dependence Matrix (GLDM), Gray Level Run Length Matrix (GLRLM) and Neighboring Gray Tone Difference Matrix (NGTDM) (**Supplemental Table 2**). A total of 1198 PET and CT radiomics features were then extracted.
3. A recursive feature elimination (29) method based on random forest (RF) algorithm was developed to delete features with minimum weight coefficient. Compared to other regularization based embedded methods like Lasso and Ridge, this random forest-based wrapper feature selection method is more convenient and more intuitive for researchers to find out the most relevant features corresponding to the predication target. Among 1198 radiomics features, that with the lowest correlation with EGFR mutation status was removed during current random forest model training iteration, and the most suitable feature sub-package was reserved for next iteration. Finally, 100 CT and 100 PET radiomics features were retained (**Supplemental Table 3**).
4. The Spearman correlation coefficient (r) was used to assess the correlation between 100 PET/CT radiomics features and four conventional PET-derived semi-quantitative parameters including SUV_max_, mean SUV (SUV_mean_), metabolic tumor volume (MTV) and total lesion glycolysis (TLG) (illustrated in **Supplemental Figure 1**). In all pair features with r > 0.85 that were highly correlated and likely to provide redundance rather than complementary information about the mutation status, the one with the lower area under the curve (AUC) by receiver operating characteristic (ROC) analysis for predicting EGFR mutation status was excluded. As a result, 54 CT and 38 PET radiomics features as well as SUV_max_ and TLG were included.
5. The univariate and multivariate (**Supplemental Tables 4, 6**) logistic regression (LR) was ultimately used to screen out the CT and PET radiomics and clinical features that can be significant to establish a robust prediction model for differentiating EGFR mutation status, and the PET/CT radiomics prediction score for EGFR mutation probability of each patient was calculated based on this model.

### Statistical Analysis

Data were analyzed using SPSS version 19.0 (IBM Corporation, Armonk, NY, USA). Spearman correlation analysis was performed to remove redundant radiomics features. Continuous data were compared using the independent samples t test. The χ^2^ test was used to compare categorical data such as patient sex. Univariate and multivariate logistic regression was used to screen out final significant variables. 10-fold cross-validation of prediction model based on selected features using machine learning algorithm of RF, support vector machine (SVM) or traditional statistics of LR were performed to test the generalization ability of the models. ROC curves were analyzed to evaluate the performance of PET/CT radiomics model for predicting EGFR mutation status. Statistical significance was set at *p* < 0.05.

## Results

### Patient Characteristics

As shown in **Table 1**, 173 patients with NSCLC were enrolled in the present study, among whom 71 (41%) tested positively for an EGFR mutation (EGFR+) and 102 (59%) were EGFR-negative (EGFR-). Female patients demonstrated a significantly higher EGFR mutation rate (64% [37/58]) than male patients (30% [34/115]). There was no statistical difference in age between patients with or without EGFR mutations. 39% (68/173) and 61% (105/173) of patients were stage I/II and stage III/IV, respectively. Seventy-one percent (122/173) of the pathological types of NSCLCs were adenocarcinoma. Among the 71 patients who were EGFR+, 38 (54%) harbored the 21 L858R mutation, 29 (41%) had the 19 del mutation, 3 (4%) had the 18 G719A substitution mutation, and 1 (1%) had the 20 T790M substitution mutation. In all clinical features, only gender was an independent and significant variable for differentiating EGFR mutation status after multivariate logistic regression analysis (**Supplemental Table 4**).

**Table 1:** Patient Characteristics.

	EGFR+	EGFR-	*p* Value
No. of patients	71	102	
Sex			
Male	34	81	<0.001
Female	37	21	
Age (y)			
Mean ± SD	60.06 ± 10.93	61.54 ± 10.89	*NS*
Range	27 ~ 86	32 ~ 83	
Clinical Stage			
I	18	28	*NS*
II	8	14	
III	19	23	
IV	26	37	
Histology			
Adenocarcinoma	60	62	0.004
Squamous cell carcinoma	8	31	
Large cell neuroendocrine carcinoma	0	4	
NSCLC-NOS	3	5	
EGFR mutation subtype			
18 G719S	3	/	
19 Del	29	/	
20 T790M	1	/	
21 L858R	38	/	

NOS, not otherwise specified; NS, not significant.

### Characteristic of Selected PET/CT Radiomics Features

Eventually, four CT and two PET radiomics features were selected to build the radiomics model based on the 173 patients, including ct_original_glszm_High Gray Level Zone Emphasis (GLSZM_HGLZE), ct_wavelet_HLL_glszm_Gray Level Non-Uniformity Normalized (GLSZM_GLNN), ct_wavelet_HLL_glszm_Zone Entropy (GLSZM_ZE), ct_exponential_gldm_Dependence Variance (GLDM_DV), pet_wavelet_LHH_firstorder_Skewness (First-order_Skewness (LHH)), pet_wavelet_LLL_firstorder_Skewness (First-order_Skewness (LLL)). The definitions of these selected radiomics features were shown in **Supplemental Table 5**. The PET/CT radiomics model prediction score for EGFR mutation probability of each patient was calculated using the following formula:

$$\begin{array}{l} \text{PET}/\text{CT}\;\text{radiomics}\;\text{model}\;\text{prediction}\;\text{score}\;=\;-6.142-2.736\\ \times \;\text{GLSZM}\_\text{HGLZE}\;+\;5.815\times \text{GLSZM}\_\text{GLNUN}\;+\;5.173\\ \times \;\text{GLSZM}\_\text{ZE}\;+\;7.737\;\times \text{GLDM}\_\text{DV}\;-\;1.734\times \;\text{First}-\text{order}\\ \_\text{Skewness }(\text{LHH})\;-\;6.142\;\times \;\text{First}-\text{order}\_\text{Skewness}\;(\text{LLL}). \end{array}$$

**Figure 2:**
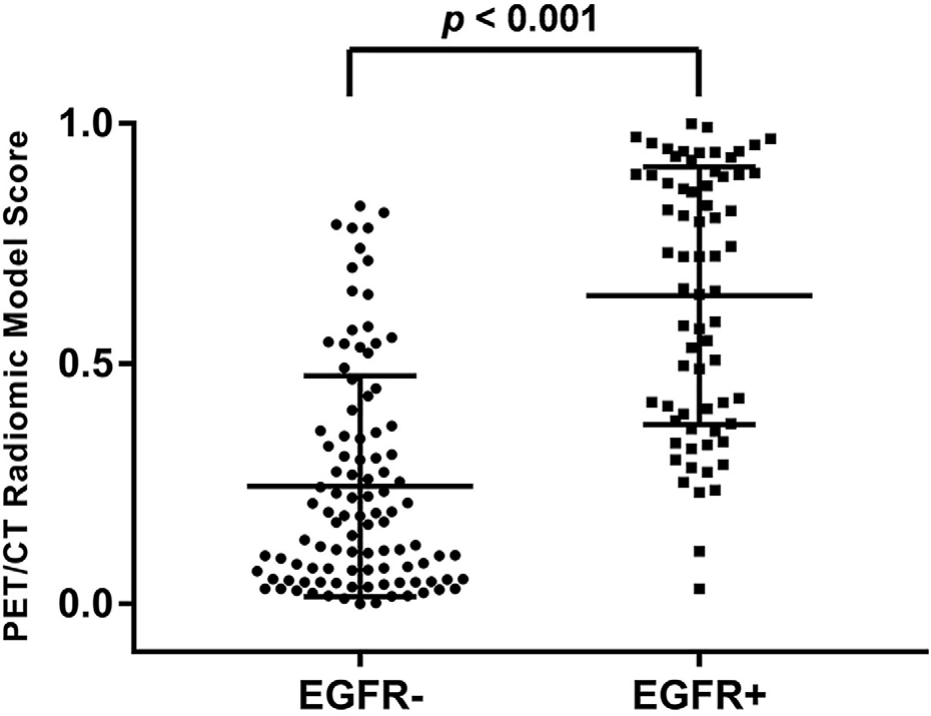
Distribution of PET/CT radiomic model prediction score of all patients. The tumors with EGFR+ had significantly higher score than those with EGFR- (*p* < 0.001).

The median and the interquartile range for selected PET/CT radiomics features and conventional PET parameters (SUV_max_ and TLG) was shown in **Table 2**. There was significant difference of every individual radiomics feature, SUV_max_ and TLG between the EGFR+ and EGFR- groups. Meanwhile, the tumors with EGFR+ had higher radiomics model score than those with EGFR- (0.722 vs. 0.170, *p* < 0.001). The PET/CT radiomics model prediction score for each patient was displayed in **Figure 2**.

**Table 2:** Characteristic of selected PET/CT radiomic features and conventional PET parameters.

Characteristic	EGFR- (N=102)	EGFR+ (N=71)	*p* Value
Conventional PET parameters			
SUV_max_	11.500 (7.070-16.950)	6.900 (4.895-10.890)	<0.001
TLG	143.181 (25.241-358.192)	33.120 (8.854-168.031)	0.018
CT Radiomic features			
GLSZM_HGLZE	0.523 (0.353-0.659)	0.314 (0.240-0.445)	<0.001
GLDM_DV	0.390 (0.248-0.501)	0.530 (0.446-0.725)	<0.001
GLSZM_GLNUN	0.286 (0.218-0.379)	0.374 (0.283-0.483)	0.001
GLSZM_ZE	0.737 (0.610-0.849)	0.631 (0.479-0.725)	<0.001
PET Radiomic features			
First-order_Skewness (LHH)	0.561 (0.392-0.764)	0.374 (0.125-0.815)	0.019
First-order_Skewness (LLL)	1.008 (0.653-1.615)	0.773 (0.537-0.982)	<0.001
PET/CT Radiomic Score	0.170 (0.051-0.359)	0.722 (0.388-0.893)	<0.001

Data were expressed as median (interquartile range).

GLSZM, Gray Level Size Zone Matrix; GLDM, Gray Level Dependence Matrix; HGLZE, High Gray Level Zone Emphasis; DV, dependence variance; GLNUN, Gray Level Non Uniformity Normalized; ZE, zone entropy; LHH and LLL are two subtypes of wavelet filters.

### Performance of the PET/CT Radiomics Model

The performance of PET/CT radiomics model was evaluated and compared with conventional PET-derived semi-quantitative parameters and clinical features for distinguishing EGFR+ from EGFR-. Both CT (AUC=0.792) and PET alone (AUC=0.738) radiomics model had better predictive performance than SUV_max_ (AUC=0.683), TLG (AUC=0.662) and gender (AUC=0.664). The AUC of PET/CT radiomics model further reached 0.868 with sensitivity of 92.8%, specificity of 66.3% and accuracy of 77.1%. Gender was only significant clinical predictor of EGFR mutation status (AUC=0.664), and used in the combined model in our study, whereas other clinical characteristics were excluded from the diagnostic model after multivariate regression analysis. The combined model, based on the PET/CT radiomics features and gender showed a comparable AUC (0.866) to PET/CT radiomics model. The sensitivity, specificity, and accuracy of different models and individual parameter in the training set were shown in **Table 3**. Subsequently, 10-fold cross-validation of the diagnostic model based on selected features using machine learning algorithm of SVM (**Table 3**), RF or traditional statistics of LR (**Supplemental Table 7**) were performed to further test the generalization ability of the models. The AUCs of PET radiomics, CT radiomics, PET/CT radiomics and combined models based on SVM were respectively 0.750, 0.754, 0.769 and 0.827.

**Table 3:** Predictive performance of EGFR mutation status using different models compared with conventional PET parameters and clinical feature.

Model/Parameters	Training set				10-fold cross validation using SVM algorithm			
	AUC	Sensitivity (%)	Specificity (%)	Accuracy (%)	AUC	Sensitivity (%)	Specificity (%)	Accuracy (%)
Combined Model	0.866	82.60%	81.20%	81.80%	0.827	73.74%	76.07%	75.29%
PET/CT Radiomics Model	0.868	92.80%	66.30%	77.10%	0.769	67.11%	67.03%	67.06%
CT Radiomics Model	0.792	58.00%	87.10%	75.30%	0.754	64.22%	69.87%	67.65%
PET Radiomics Model	0.738	55.10%	82.20%	71.20%	0.750	60.29%	69.69%	67.06%
Gender	0.664	53.60%	79.20%	68.80%	/	/	/	/
SUV_max_	0.683	84.10%	49.50%	63.50%	/	/	/	/
TLG	0.662	66.70%	64.40%	65.30%	/	/	/	/

SVM, support vector machine.

In addition, we tried to investigate the possibility of radiomics features for discriminating two main mutation subtypes (**Table 4**). As previous reported, there was no difference of SUV_max_ or TLG between the 19 del and the 21 L858R mutation group. In all radiomics features, only one PET radiomics feature (pet_logarithm_glcm_Difference Variance, GLCM_DV) was significantly predictive (AUC=0.661) for differentiating these two mutation subtypes. However, it had low accuracy (43.1%) for the prediction of EGFR mutation subtypes.

**Table 4:** Predictive performance of EGFR mutation subtypes using PET/CT radiomic features compared with conventional PET parameters.

Parameters/Feature	21 L858R mutation(N =38)	19 Del mutation(N=29)	*p* Value	AUC	Sensitivity(%)	Specificity(%)	Accuracy(%)
SUV_max_	7.5 (5.355-11.650)	6.695 (4.450-10.450)	0.134	/	/	/	/
TLG	37.98 (12.703-180.620)	26.014 (4.529-164.770)	0.408	/	/	/	/
GLCM_DV	1134.093 (801.011-1667.094)	808.42 (433.669-1353.409)	0.016	0.661	75.70%	57.10%	43.10%

Data were expressed as median (interquartile range).

GLCM, Gray Level Co-occurrence Matrix; DV, dependence variance.

## Discussion

EGFR-TKI is an important treatment for patients with NSCLC. When treated with TKI, patients with EGFR mutations experience significantly longer survival than those with wild-type EGFR. As such, identification of EGFR mutation status is crucial for TKI treatment to be effective; however, the molecular test for EGFR mutation status sometimes cannot be performed when a tumor sample is not available.

Although a significant correlation between the tumor glucose metabolism level captured on PET images and EGFR mutation status has been found in multiple previous studies (11–14), namely lower SUV_max_ in NSCLCs with EGFR mutation than those with wild type EGFR, conventional PET-derived semi-quantitative parameters didn’t show enough satisfactory predictive ability to be applied in clinical practice. Consistent with previous studies, SUV_max_ as a single pixel value only showed moderate AUC for distinguishing mutant EGFR from wild type in our study, whereas total lesion glycolysis (TLG) as a volumetric measurement of tumor glucose metabolism showed no higher predictive performance either. Therefore, our present study established a model based on ^18^FDG PET/CT radiomics to improve the predictive performance for EGFR mutation status in patients with NSCLC.

In our study, four CT radiomics features and two PET features were selected to establish the predictive model with significantly higher AUC than that of SUV_max_ and TLG. Among these selected radiomics features, GLSZM_HGLZE from CT images measures the distribution of the higher gray-level values with a higher value indicating larger high-density areas proportion in tumor, which suggested that the tumors with EGFR+ had lower density than the EGFR- group in our study. In agreement with our finding, more ground-glass opacity and less solid components were observed in lung cancers with EGFR mutation (30) with lower mean CT values when compared to those with wild-type EGFR (31). The remaining 5 radiomics features, including three CT features (GLDM_DV, GLSZM_GLNN, GLSZM_ZE) and two PET features (First-order_Skewness (LHH), First-order_Skewness (LLL)), are all related to image uniformity and heterogeneity. In our study, the EGFR+ group was more heterogeneous on both PET and CT images than the EGFR- group. Our findings were similar to previous studies (21–23). They found that those image texture feature measuring the variability of gray-level intensity or the asymmetry of the distribution of gray-level values were significant predictive of EGFR mutation status. In summary, the NSCLCs with EGFR mutation had lower glucose metabolism and density, with more heterogeneity on both PET and CT images than those with wild-type EGFR. Owing to the bi-modal image features, PET/CT radiomics model in recent studies (0.79 in Zhang J’s study (23); 0.80 in Li X’s study (22); 0.77 in our study) has showed higher AUC than those generated by PET (0.67 in Yip, SS’s study (21)) or CT (0.69 in Rios Velazquez, E’s study (19); 0.56-0.75 in Sacconi, B’s study (31)) radiomics features alone for predicting EGFR mutation status. However, compared with larger sample size in CT radiomics research, the current sample size in PET/CT radiomics-related studies is generally limited, and thus the generalization ability of PET/CT radiomics-based model remains to be further tested.

Clinical features in patients with NSCLC are also non-negligible variables in the evaluation of EGFR mutations, which are more likely to occur in Asians, adenocarcinomas, females, and nonsmokers (32). In our study, gender was only significant clinical predictor of EGFR mutation status. Smoking history was not included in our study due to the complexity of its definition, including the length of history, whether to quit or repeat smoking, etc. This complexity of smoking history made the simple classification of yes or no meaningless. Gender as only clinical characteristic was selected in combine model of our study. The addition of clinical characteristics to PET/CT radiomics model, to varying degrees, increase the diagnostic performance of diagnostic model in previous studies (22, 23) and our study, which finally reached 82.6% of diagnostic accuracy in Li X’s study (22), 80.0% in Zhang J’s study (23), and 75.3% in our study for predicting EGFR mutation status. It suggested that the combined model might be an alternative indicator of EGFR mutations when tissue samples are not available.

The 19 del and 21 L858R mutations are the main two EGFR mutation subtypes. Although both mutation subtypes are sensitive to EGFR-TKI treatment, it is now being recognized that patients with the 19 del mutation experience better clinical outcomes compared to those with the 21 L858R mutation (33, 34). Similar to a previous study investigating a large cohort of Chinese patients (14), we found that SUV_max_ or TLG had no ability to classify the 19 del and the 21 L858R mutation. We tried to investigate the possibility of PET/CT radiomics features for distinguishing these two subtypes. Only GLCM_DV from PET images, which measures the heterogeneity of different intensity-level matrix, showed significant but unsatisfactory predictive performance in our study (AUC=0.661). Liu Q, et al. recent study (35) established a predictive model for EGFR mutation subtypes using machine learning algorithm, which seemed to have better classification performance (AUC=0.77 and 0.92 for respectively predicting exons 19 del and 21 L858R mutations) than ours. However, the number of exons 19 del and 21 L858R mutations was small in Liu Q’s study (only 44 and 31 cases respectively), especially when divided as the train and test cohorts, so the generalization ability of the predictive model was not clear.

The limited number of cases was one of the main factors restricting our research to obtain more reliable conclusions. Larger-scale data based on multi-center may be a solution in our next research. However, multi-center study may bring another important issue that affects the generalization ability of the model, that is, the variable PET imaging protocols among multi-centers including image acquisition and reconstruction conditions will be unable to ensure the uniformity and comparability of extracted radiomics features, thus affecting the sensitivity and specificity of radiomics model. Papp L, et al. suggested that larger matrix size/smaller voxel size, point-spread function reconstruction algorithms, and narrow Gaussian post-filtering helped minimize feature variations (36). The variability of PET radiomics is also feature-dependent. GLCM and shape features are the least sensitive to PET imaging system variations (36, 37). Although the single center study maintains the image acquisition and reconstruction methods consistent in all enrolled patients, thus avoiding the influence of the above-described factors as much as possible, the standardization of large databases from multi-centers will remain an unavoidable key step in further research.

In conclusion, EGFR mutations status in patients with NSCLC could be well predicted by the model based on ^18^F-FDG PET/CT radiomics and clinical features, providing an alternative useful method for the selection of TKI therapy.

## Data Availability Statement

All datasets presented in this study are included in the article/**Supplementary Material**.

## Ethics Statement

The studies involving human participants were reviewed and approved by Ruijin Hospital Ethics Committee Shanghai Jiao Tong University School of Medicine. The patients/participants provided their written informed consent to participate in this study.

## Author Contributions

Conceptualization: MZ, CS, DQ, and BL. Data curation: JL. Formal analysis: YB, WR, XL, MZ, and XH. Investigation: MZ, YZ, QQ, and HM. Methodology: YB and JX. Project administration: MZh and BL. Supervision: DQ and BL. Writing—original draft: MZ and YB. Writing—review and editing: DQ and BL. All authors contributed to the article and approved the submitted version.

## Funding

This research was supported by grants from the National Key Research and Development Program of China (2018YFC0116402), National Natural Science Foundation of China (81974276), Shanghai Jiao Tong University Med-X Interdisciplinary Research Funding (YG2017MS61), Shanghai Pujiang Program (18PJD030) and Shanghai Municipal Key Clinical Specialty (shslczdzk03403).

## Conflict of Interest

The authors declare that the research was conducted in the absence of any commercial or financial relationships that could be construed as a potential conflict of interest.
