# Supplementary material for: Performance of 18F-FDG PET/CT Radiomics for Predicting EGFR Mutation Status in Patients With Non-Small Cell Lung Cancer
Source: Front Oncol. 2020 Oct 8;10:568857. doi: 10.3389/fonc.2020.568857 (PMC7578399; doi:10.3389/fonc.2020.568857)
Supplement: Supplementary file 1 [file DataSheet_1.zip › Revised supplementary tables.docx]

**Supplemental Table 1. Descriptions of filters types**

| Filters | Descriptions |
| --- | --- |
| Laplacian of Gaussian (LoG) | Emphasize the edges and rapidly changed intensities |
| Wavelet | Use low-pass or high-pass filters in 3D image including HHH, HHL, HLH, HLL, LHH, LHL, LLH and LLL |
| Square | Compute the square of image intensities |
| Square Root | Compute the square root of the absolute value of image intensities |
| Logarithm | Compute the logarithm of the absolute value of the original intensities, then add the result with 1 |
| Exponential | Compute the exponential of the original image |

**Supplemental Table 2. Descriptions of image feature types**

| Feature types |  | Descriptions |
| --- | --- | --- |
| First Order Statistics |  | Describe the distribution of voxel’s gray intensities within the image region defined by segmentation via analyzing gray histogram. For example, Energy, Entropy, Maximum, Minimum, Uniformity, Skewness, etc. |
| Shape |  | Descriptors of the 3D shape and size of the ROI. These features are independent from the gray level of image. Hence, they are only extracted from original and non-derived image. They include Volume, Surface area, Maximum 3D diameter, Flatness, etc. |
| Gray Level Co-occurrence Matrix (GLCM) |  | Describes the second-order joint probability function of masked region. Features derived from a GLCM contain Correlation, Difference Variance, Inverse Difference Moment (IDM), etc. |
| Gray Level Size Zone Matrix (GLSZM) |  | Considers the connected voxel with the same gray level intensity. It should be mentioned that GLSZM is independent of rotation and direction. Features derived from a GLSZM contain Gray Level Non-Uniformity (GLN), Gray Level Variance, High Gray Zone Emphasis, etc. |
| Gray Level Dependence Matrix (GLDM) |  | Describe the compactness of voxels. Elements in matric reflect the dependence of neighboring voxels. Features derived from a GLDM contain Large Dependent Emphasis, Gray Level Non-Uniformity, Dependent Variance, etc. |
| Gray Level Run Length Matrix (GLRLM) |  | Describes the run length of same gray level in given angle. It is a matric reflects high-order texture characteristics of image in ROI. Features derived from a GLRLM contain Gray Level Non-Uniformity (GLN), Run Variance (RV), High Gray Level Run Emphasis, etc. |
| Neighboring Gray Tone Difference Matrix (NGTDM) |  | Quantifies the difference between a voxel’s gray level and the average of its neighbors’ gray level. Features derived from a NGTDM contain Complexity, Strength, Contrast, etc. |

**Supplemental Table 3** List of 100 radiomic features and four conventional PET-derived parameters

| Filter Type | Features/Parameter |
| --- | --- |
| / | SUV_max_ |
|  | SUV_mean_ |
|  | MTV |
|  | TLG |
| Original | original_shape_Maximum2DDiameterRow |
|  | original_shape_Maximum3DDiameter |
|  | original_shape_Sphericity |
|  | original_shape_SurfaceArea |
|  | original_firstorder_Skewness |
|  | original_glcm_Correlation |
|  | original_glcm_Idmn |
|  | original_glcm_Idn |
|  | original_glrlm_GrayLevelNonUniformity |
|  | original_glszm_GrayLevelNonUniformity |
|  | original_gldm_GrayLevelNonUniformity |
| Wavelet | LLH_firstorder_Kurtosis |
|  | LLH_glcm_Idm |
|  | LLH_glrlm_RunLengthNonUniformityNormalized |
|  | LLH_glrlm_RunVariance |
|  | LLH_glszm_GrayLevelNonUniformity |
|  | LHL_firstorder_Skewness |
|  | LHL_glcm_Idmn |
|  | LHL_glcm_Idn |
|  | LHL_glrlm_GrayLevelNonUniformity |
|  | LHL_glszm_GrayLevelNonUniformity |
|  | LHL_gldm_GrayLevelNonUniformity |
|  | LHH_firstorder_Mean |
|  | LHH_firstorder_Median |
|  | LHH_firstorder_Skewness |
|  | LHH_glcm_Correlation |
|  | LHH_glszm_GrayLevelNonUniformity |
|  | LHH_glszm_ZoneVariance |
|  | HLL_firstorder_Median |
|  | HLL_firstorder_Skewness |
|  | HLL_glszm_GrayLevelNonUniformity |
|  | HLL_gldm_GrayLevelNonUniformity |
|  | HLH_firstorder_Kurtosis |
|  | HLH_firstorder_Median |
|  | HLH_glcm_Correlation |
|  | HLH_glszm_GrayLevelNonUniformity |
|  | HHL_firstorder_Kurtosis |
|  | HHL_firstorder_Mean |
|  | HHL_firstorder_Skewness |
|  | HHL_glszm_GrayLevelNonUniformity |
|  | HHH_firstorder_RobustMeanAbsoluteDeviation |
|  | HHH_glcm_Imc1 |
|  | HHH_glrlm_GrayLevelNonUniformity |
|  | HHH_glrlm_RunPercentage |
|  | HHH_glszm_GrayLevelNonUniformity |
|  | HHH_glszm_SizeZoneNonUniformityNormalized |
|  | HHH_glszm_SmallAreaEmphasis |
|  | HHH_glszm_ZonePercentage |
|  | HHH_gldm_SmallDependenceEmphasis |
|  | LLL_firstorder_Kurtosis |
|  | LLL_firstorder_Skewness |
|  | LLL_glcm_Correlation |
|  | LLL_glcm_Imc1 |
|  | LLL_glcm_Idm |
|  | LLL_glcm_Idn |
|  | LLL_glrlm_RunPercentage |
|  | LLL_glszm_GrayLevelNonUniformity |
|  | LLL_glszm_SmallAreaEmphasis |
| Square | square_firstorder_Skewness |
|  | square_glcm_Idmn |
|  | square_glcm_Idn |
|  | square_glrlm_LongRunEmphasis |
|  | square_glrlm_LongRunLowGrayLevelEmphasis |
|  | square_glrlm_LowGrayLevelRunEmphasis |
|  | square_glrlm_RunLengthNonUniformityNormalized |
|  | square_glrlm_RunPercentage |
|  | square_glrlm_RunVariance |
|  | square_glrlm_ShortRunLowGrayLevelEmphasis |
|  | square_glszm_GrayLevelNonUniformity |
|  | square_glszm_SmallAreaLowGrayLevelEmphasis |
|  | square_glszm_ZonePercentage |
|  | square_gldm_DependenceVariance |
|  | square_gldm_LargeDependenceEmphasis |
|  | square_gldm_LowGrayLevelEmphasis |
|  | square_gldm_SmallDependenceLowGrayLevelEmphasis |
| Squareroot | squareroot_firstorder_Skewness |
|  | squareroot_glcm_Idn |
|  | squareroot_glrlm_RunLengthNonUniformity |
|  | squareroot_glszm_GrayLevelNonUniformity |
|  | squareroot_gldm_LargeDependenceLowGrayLevelEmphasis |
| Logarithm | logarithm_glcm_Correlation |
|  | logarithm_glcm_DifferenceAverage |
|  | logarithm_glcm_DifferenceVariance |
|  | logarithm_glcm_Idm |
|  | logarithm_glcm_Idn |
|  | logarithm_glrlm_ShortRunEmphasis |
|  | logarithm_glszm_GrayLevelNonUniformity |
|  | logarithm_glszm_LowGrayLevelZoneEmphasis |
|  | logarithm_glszm_SizeZoneNonUniformity |
|  | logarithm_gldm_DependenceNonUniformity |
|  | logarithm_gldm_DependenceVariance |
|  | logarithm_ngtdm_Strength |
| Exponential | exponential_firstorder_Kurtosis |
|  | exponential_glrlm_GrayLevelNonUniformity |
|  | exponential_glrlm_LongRunHighGrayLevelEmphasis |
|  | exponential_glrlm_RunPercentage |
|  | exponential_glszm_SizeZoneNonUniformity |
|  | exponential_glszm_SmallAreaEmphasis |
|  | exponential_glszm_SmallAreaHighGrayLevelEmphasis |
|  | exponential_glszm_SmallAreaLowGrayLevelEmphasis |

**Supplemental Table 4** Univariate and multivariate logistic regression analysis of clinical informations, conventional PET parameters and PET/CT radiomic features for differentiating EGFR mutation status

| Variables | Univariate analysis | Multivariate  analysis | | |
| --- | --- | --- | --- | --- |
|  | p | p | OR | 95% CI |
| Stage | 0.940 | 0.058 |  |  |
| Pathology | 0.002 | 0.531 |  |  |
| Age | 0.279 | 0.581 |  |  |
| Gender | 0.000 | 0.001 | 0.247 | 0.104-0.583 |
| SUVmax | 0.000 | 0.342 |  |  |
| TLG | 0.018 | 0.234 |  |  |
| ct_original_shape_Sphericity | 0.159 | 0.187 |  |  |
| ct_original_glcm_DifferenceVariance | 0.443 | 0.897 |  |  |
| ct_original_glszm_HighGrayLevelZoneEmphasis | 0.000 | 0.045 | 0.069 | 0.005-0.940 |
| ct_original_glszm_SmallAreaLowGrayLevelEmphasis | 0.021 | 0.522 |  |  |
| ct_original_gldm_LargeDependenceLowGrayLevelEmphasis | 0.467 | 0.472 |  |  |
| ct_wavelet_LLH_firstorder_Kurtosis | 0.016 | 0.674 |  |  |
| ct_wavelet_LLH_firstorder_Mean | 0.137 | 0.763 |  |  |
| ct_wavelet_LLH_firstorder_Range | 0.001 | 0.736 |  |  |
| ct_wavelet_LLH_glcm_Idmn | 0.001 | 0.561 |  |  |
| ct_wavelet_LLH_glrlm_HighGrayLevelRunEmphasis | 0.290 | 0.841 |  |  |
| ct_wavelet_LLH_gldm_DependenceEntropy | 0.707 | 0.386 |  |  |
| ct_wavelet_LLH_ngtdm_Busyness | 0.011 | 0.566 |  |  |
| ct_wavelet_LHL_firstorder_Mean | 0.512 | 0.018 |  |  |
| ct_wavelet_LHL_firstorder_Range | 0.001 | 0.598 |  |  |
| ct_wavelet_LHL_glcm_ClusterShade | 0.366 | 0.911 |  |  |
| ct_wavelet_LHL_glszm_LowGrayLevelZoneEmphasis | 0.066 | 0.570 |  |  |
| ct_wavelet_LHL_glszm_ZoneEntropy | 0.005 | 0.527 |  |  |
| ct_wavelet_LHL_gldm_LargeDependenceHighGrayLevelEmphasis | 0.000 | 0.833 |  |  |
| ct_wavelet_LHH_firstorder_Skewness | 0.477 | 0.168 |  |  |
| ct_wavelet_LHH_glszm_SizeZoneNonUniformityNormalized | 0.641 | 0.564 |  |  |
| ct_wavelet_LHH_gldm_DependenceVariance | 0.705 | 0.198 |  |  |
| ct_wavelet_LHH_gldm_SmallDependenceLowGrayLevelEmphasis | 0.011 | 0.757 |  |  |
| ct_wavelet_HLL_firstorder_90Percentile | 0.398 | 0.121 |  |  |
| ct_wavelet_HLL_firstorder_Maximum | 0.000 | 0.526 |  |  |
| ct_wavelet_HLL_firstorder_TotalEnergy | 0.000 | 0.454 |  |  |
| ct_wavelet_HLL_glcm_ClusterShade | 0.156 | 0.764 |  |  |
| ct_wavelet_HLL_glcm_Correlation | 0.727 | 0.363 |  |  |
| ct_wavelet_HLL_glszm_GrayLevelNonUniformityNormalized | 0.001 | 0.002 | 335.243 | 7.976-14090.936 |
| ct_wavelet_HLL_glszm_ZoneEntropy | 0.000 | 0.004 | 176.391 | 5.281-5891.991 |
| ct_wavelet_HHL_firstorder_Mean | 0.063 | 0.736 |  |  |
| ct_wavelet_HHH_glcm_Idmn | 0.006 | 0.701 |  |  |
| ct_wavelet_HHH_glrlm_RunEntropy | 0.000 | 0.845 |  |  |
| ct_wavelet_HHH_glszm_GrayLevelNonUniformityNormalized | 0.006 | 0.812 |  |  |
| ct_wavelet_HHH_glszm_GrayLevelVariance | 0.024 | 0.658 |  |  |
| ct_wavelet_HHH_glszm_LargeAreaHighGrayLevelEmphasis | 0.011 | 0.925 |  |  |
| ct_wavelet_HHH_gldm_DependenceVariance | 0.000 | 0.434 |  |  |
| ct_wavelet_HHH_gldm_LargeDependenceHighGrayLevelEmphasis | 0.002 | 0.953 |  |  |
| ct_wavelet_LLL_gldm_LargeDependenceLowGrayLevelEmphasis | 0.527 | 0.800 |  |  |
| ct_wavelet_LLL_ngtdm_Complexity | 0.075 | 0.951 |  |  |
| ct_wavelet_LLL_ngtdm_Contrast | 0.001 | 0.668 |  |  |
| ct_square_firstorder_Range | 0.014 | 0.612 |  |  |
| ct_square_glcm_Imc1 | 0.161 | 0.491 |  |  |
| ct_square_glszm_SmallAreaEmphasis | 0.002 | 0.774 |  |  |
| ct_square_gldm_DependenceVariance | 0.292 | 0.714 |  |  |
| ct_square_ngtdm_Busyness | 0.004 | 0.710 |  |  |
| ct_squareroot_glszm_LowGrayLevelZoneEmphasis | 0.001 | 0.305 |  |  |
| ct_logarithm_glcm_Idn | 0.311 | 0.954 |  |  |
| ct_logarithm_glrlm_GrayLevelNonUniformity | 0.002 | 0.868 |  |  |
| ct_exponential_firstorder_Minimum | 0.018 | 0.414 |  |  |
| ct_exponential_firstorder_Variance | 0.153 | 0.354 |  |  |
| ct_exponential_glrlm_GrayLevelNonUniformity | 0.002 | 0.956 |  |  |
| ct_exponential_glrlm_RunLengthNonUniformity | 0.990 | 0.771 |  |  |
| ct_exponential_glrlm_RunLengthNonUniformityNormalized | 0.000 | 0.408 |  |  |
| ct_exponential_gldm_DependenceVariance | 0.000 | 0.000 | 433.022 | 20.873-8983.267 |
| pet_original_shape_Sphericity | 0.005 | 0.584 |  |  |
| pet_original_glcm_Idmn | 0.000 | 0.223 |  |  |
| pet_wavelet_LLH_firstorder_Kurtosis | 0.000 | 0.803 |  |  |
| pet_wavelet_LHL_firstorder_Skewness | 0.005 | 0.695 |  |  |
| pet_wavelet_LHH_firstorder_Mean | 0.212 | 0.601 |  |  |
| pet_wavelet_LHH_firstorder_Median | 0.115 | 0.588 |  |  |
| pet_wavelet_LHH_firstorder_Skewness | 0.019 | 0.004 | 0.163 | 0.047-0.565 |
| pet_wavelet_LHH_glcm_Correlation | 0.396 | 0.686 |  |  |
| pet_wavelet_HLL_firstorder_Median | 0.070 | 0.440 |  |  |
| pet_wavelet_HLL_firstorder_Skewness | 0.001 | 0.534 |  |  |
| pet_wavelet_HLH_firstorder_Kurtosis | 0.004 | 0.395 |  |  |
| pet_wavelet_HLH_firstorder_Median | 0.596 | 0.271 |  |  |
| pet_wavelet_HLH_glcm_Correlation | 0.519 | 0.576 |  |  |
| pet_wavelet_HHL_firstorder_Kurtosis | 0.000 | 0.834 |  |  |
| pet_wavelet_HHL_firstorder_Mean | 0.395 | 0.627 |  |  |
| pet_wavelet_HHL_firstorder_Skewness | 0.031 | 0.082 |  |  |
| pet_wavelet_HHH_firstorder_RobustMeanAbsoluteDeviation | 0.216 | 0.183 |  |  |
| pet_wavelet_HHH_glcm_Imc1 | 0.000 | 0.937 |  |  |
| pet_wavelet_HHH_glrlm_GrayLevelNonUniformity | 0.002 | 0.387 |  |  |
| pet_wavelet_HHH_glszm_GrayLevelNonUniformity | 0.000 | 0.661 |  |  |
| pet_wavelet_HHH_glszm_SizeZoneNonUniformityNormalized | 0.013 | 0.622 |  |  |
| pet_wavelet_HHH_glszm_ZonePercentage | 0.063 | 0.548 |  |  |
| pet_wavelet_LLL_firstorder_Skewness | 0.000 | 0.026 | 0.395 | 0.174-0.896 |
| pet_wavelet_LLL_glcm_Imc1 | 0.001 | 0.754 |  |  |
| pet_square_glrlm_RunVariance | 0.033 | 0.273 |  |  |
| pet_square_glszm_GrayLevelNonUniformity | 0.001 | 0.915 |  |  |
| pet_square_gldm_DependenceVariance | 0.005 | 0.206 |  |  |
| pet_square_gldm_LowGrayLevelEmphasis | 0.510 | 0.419 |  |  |
| pet_square_gldm_SmallDependenceLowGrayLevelEmphasis | 0.155 | 0.457 |  |  |
| pet_squareroot_firstorder_Skewness | 0.005 | 0.782 |  |  |
| pet_logarithm_glcm_DifferenceVariance | 0.038 | 0.387 |  |  |
| pet_logarithm_gldm_DependenceVariance | 0.373 | 0.799 |  |  |
| pet_logarithm_ngtdm_Strength | 0.285 | 0.306 |  |  |
| pet_exponential_firstorder_Kurtosis | 0.014 | 0.568 |  |  |
| pet_exponential_glrlm_LongRunHighGrayLevelEmphasis | 0.069 | 0.370 |  |  |
| pet_exponential_glrlm_RunPercentage | 0.005 | 0.721 |  |  |
| pet_exponential_glszm_SmallAreaEmphasis | 0.020 | 0.356 |  |  |
| pet_exponential_glszm_SmallAreaLowGrayLevelEmphasis | 0.318 | 0.111 |  |  |

**Supplemental Table 5.** Description of selected four CT and two PET radiomic featurs

| Features | Description |
| --- | --- |
| CT Radiomic features |  |
| GLSZM_HGLZE | Measures the distribution of the higher gray-level values in the image. |
| GLDM_DV | Measures the variance in dependence size in the image. |
| GLSZM_GLNN | Measures the variability of gray-level intensity values in the image. This is the normalized version of the GLN formula. |
| GLSZM_ZE | Measures the uncertainty/randomness in the distribution of zone sizes and gray levels. |
|  |  |
| PET Radiomic features |  |
| First-order_Skewness (LHH) | Measures the asymmetry of the distribution of values about the mean value. |
| First-order_Skewness (LLL) |  |

ct_original_glszm_High Gray Level Zone Emphasis: GLSZM_HGLZE;

ct_wavelet_HLL_glszm_Gray Level Non-Uniformity Normalized: GLSZM_GLNN;

ct_wavelet_HLL_glszm_Zone Entropy: GLSZM_ZE;

ct_exponential_gldm_Dependence Variance: GLDM_DV;

pet_wavelet_LHH_firstorder_Skewness: First-order_Skewness (LHH);

pet_wavelet_LLL_firstorder_Skewness: First-order_Skewness (LLL)

**Supplemental Table 6.** Univariate and multivariate logistic regression analysis of clinical informations, conventional PET parameters and PET/CT radiomic features for differentiating the 19 del from the 21 L858R mutation**.**

| Variables | Univariate analysis | | Multivariate analysis | | | | |
| --- | --- | --- | --- | --- | --- | --- | --- |
|  | p | p | | | OR | 95% CI |  |
| Stage | 0.093 | |  |  |  | |  |
| Pathology | 0.817 | |  |  |  | |  |
| Age | 0.314 | |  |  |  | |  |
| Gender | 0.447 | |  |  |  | |  |
| SUVmax | 0.134 | |  |  |  | |  |
| TLG | 0.408 | |  |  |  | |  |
| ct_original_shape_Sphericity | 0.064 | |  |  |  | |  |
| ct_original_glcm_DifferenceVariance | 0.736 | |  |  |  | |  |
| ct_original_glszm_HighGrayLevelZoneEmphasis | 0.443 | |  |  |  | |  |
| ct_original_glszm_SmallAreaLowGrayLevelEmphasis | 0.119 | |  |  |  | |  |
| ct_original_gldm_LargeDependenceLowGrayLevelEmphasis | 0.343 | |  |  |  | |  |
| ct_wavelet_LLH_firstorder_Kurtosis | 0.466 | |  |  |  | |  |
| ct_wavelet_LLH_firstorder_Mean | 0.482 | |  |  |  | |  |
| ct_wavelet_LLH_firstorder_Range | 0.372 | |  |  |  | |  |
| ct_wavelet_LLH_glcm_Idmn | 0.084 | |  |  |  | |  |
| ct_wavelet_LLH_glrlm_HighGrayLevelRunEmphasis | 0.691 | |  |  |  | |  |
| ct_wavelet_LLH_gldm_DependenceEntropy | 0.048 | |  |  |  | |  |
| ct_wavelet_LLH_ngtdm_Busyness | 0.924 | |  |  |  | |  |
| ct_wavelet_LHL_firstorder_Mean | 0.888 | |  |  |  | |  |
| ct_wavelet_LHL_firstorder_Range | 0.043 | |  |  |  | |  |
| ct_wavelet_LHL_glcm_ClusterShade | 0.318 | |  |  |  | |  |
| ct_wavelet_LHL_glszm_LowGrayLevelZoneEmphasis | 0.971 | |  |  |  | |  |
| ct_wavelet_LHL_glszm_ZoneEntropy | 0.224 | |  |  |  | |  |
| ct_wavelet_LHL_gldm_LargeDependenceHighGrayLevelEmphasis | 0.011 | |  |  |  | |  |
| ct_wavelet_LHH_firstorder_Skewness | 0.345 | |  |  |  | |  |
| ct_wavelet_LHH_glszm_SizeZoneNonUniformityNormalized | 0.485 | |  |  |  | |  |
| ct_wavelet_LHH_gldm_DependenceVariance | 0.161 | |  |  |  | |  |
| ct_wavelet_LHH_gldm_SmallDependenceLowGrayLevelEmphasis | 0.052 | |  |  |  | |  |
| ct_wavelet_HLL_firstorder_90Percentile | 0.930 | |  |  |  | |  |
| ct_wavelet_HLL_firstorder_Maximum | 0.345 | |  |  |  | |  |
| ct_wavelet_HLL_firstorder_TotalEnergy | 0.097 | |  |  |  | |  |
| ct_wavelet_HLL_glcm_ClusterShade | 0.328 | |  |  |  | |  |
| ct_wavelet_HLL_glcm_Correlation | 0.969 | |  |  |  | |  |
| ct_wavelet_HLL_glszm_GrayLevelNonUniformityNormalized | 0.212 | |  |  |  | |  |
| ct_wavelet_HLL_glszm_ZoneEntropy | 0.033 | |  |  |  | |  |
| ct_wavelet_HHL_firstorder_Mean | 0.411 | |  |  |  | |  |
| ct_wavelet_HHH_glcm_Idmn | 0.015 | |  |  |  | |  |
| ct_wavelet_HHH_glrlm_RunEntropy | 0.016 | |  |  |  | |  |
| ct_wavelet_HHH_glszm_GrayLevelNonUniformityNormalized | 0.701 | |  |  |  | |  |
| ct_wavelet_HHH_glszm_GrayLevelVariance | 0.012 | |  |  |  | |  |
| ct_wavelet_HHH_glszm_LargeAreaHighGrayLevelEmphasis | 0.641 | |  |  |  | |  |
| ct_wavelet_HHH_gldm_DependenceVariance | 0.541 | |  |  |  | |  |
| ct_wavelet_HHH_gldm_LargeDependenceHighGrayLevelEmphasis | 0.009 | |  |  |  | |  |
| ct_wavelet_LLL_gldm_LargeDependenceLowGrayLevelEmphasis | 0.231 | |  |  |  | |  |
| ct_wavelet_LLL_ngtdm_Complexity | 0.051 | |  |  |  | |  |
| ct_wavelet_LLL_ngtdm_Contrast | 0.154 | |  |  |  | |  |
| ct_square_firstorder_Range | 0.297 | |  |  |  | |  |
| ct_square_glcm_Imc1 | 0.655 | |  |  |  | |  |
| ct_square_glszm_SmallAreaEmphasis | 0.011 | |  |  |  | |  |
| ct_square_gldm_DependenceVariance | 0.359 | |  |  |  | |  |
| ct_square_ngtdm_Busyness | 0.106 | |  |  |  | |  |
| ct_squareroot_glszm_LowGrayLevelZoneEmphasis | 0.197 | |  |  |  | |  |
| ct_logarithm_glcm_Idn | 0.881 | |  |  |  | |  |
| ct_logarithm_glrlm_GrayLevelNonUniformity | 0.799 | |  |  |  | |  |
| ct_exponential_firstorder_Minimum | 0.637 | |  |  |  | |  |
| ct_exponential_firstorder_Variance | 0.071 | |  |  |  | |  |
| ct_exponential_glrlm_GrayLevelNonUniformity | 0.269 | |  |  |  | |  |
| ct_exponential_glrlm_RunLengthNonUniformity | 0.377 | |  |  |  | |  |
| ct_exponential_glrlm_RunLengthNonUniformityNormalized | 0.019 | |  |  |  | |  |
| ct_exponential_gldm_DependenceVariance | 0.553 | |  |  |  | |  |
| pet_original_shape_Sphericity | 0.794 | |  |  |  | |  |
| pet_original_glcm_Idmn | 0.144 | |  |  |  | |  |
| pet_wavelet_LLH_firstorder_Kurtosis | 0.847 | |  |  |  | |  |
| pet_wavelet_LHL_firstorder_Skewness | 0.551 | |  |  |  | |  |
| pet_wavelet_LHH_firstorder_Mean | 0.519 | |  |  |  | |  |
| pet_wavelet_LHH_firstorder_Median | 0.250 | |  |  |  | |  |
| pet_wavelet_LHH_firstorder_Skewness | 0.503 | |  |  |  | |  |
| pet_wavelet_LHH_glcm_Correlation | 0.055 | |  |  |  | |  |
| pet_wavelet_HLL_firstorder_Median | 0.573 | |  |  |  | |  |
| pet_wavelet_HLL_firstorder_Skewness | 0.295 | |  |  |  | |  |
| pet_wavelet_HLH_firstorder_Kurtosis | 0.587 | |  |  |  | |  |
| pet_wavelet_HLH_firstorder_Median | 0.263 | |  |  |  | |  |
| pet_wavelet_HLH_glcm_Correlation | 0.176 | |  |  |  | |  |
| pet_wavelet_HHL_firstorder_Kurtosis | 0.812 | |  |  |  | |  |
| pet_wavelet_HHL_firstorder_Mean | 0.529 | |  |  |  | |  |
| pet_wavelet_HHL_firstorder_Skewness | 0.388 | |  |  |  | |  |
| pet_wavelet_HHH_firstorder_RobustMeanAbsoluteDeviation | 0.839 | |  |  |  | |  |
| pet_wavelet_HHH_glcm_Imc1 | 0.175 | |  |  |  | |  |
| pet_wavelet_HHH_glrlm_GrayLevelNonUniformity | 0.368 | |  |  |  | |  |
| pet_wavelet_HHH_glszm_GrayLevelNonUniformity | 0.614 | |  |  |  | |  |
| pet_wavelet_HHH_glszm_SizeZoneNonUniformityNormalized | 0.489 | |  |  |  | |  |
| pet_wavelet_HHH_glszm_ZonePercentage | 0.734 | |  |  |  | |  |
| pet_wavelet_LLL_firstorder_Skewness | 0.028 | |  |  |  | |  |
| pet_wavelet_LLL_glcm_Imc1 | 0.158 | |  |  |  | |  |
| pet_square_glrlm_RunVariance | 0.863 | |  |  |  | |  |
| pet_square_glszm_GrayLevelNonUniformity | 0.908 | |  |  |  | |  |
| pet_square_gldm_DependenceVariance | 0.572 | |  |  |  | |  |
| pet_square_gldm_LowGrayLevelEmphasis | 0.194 | |  |  |  | |  |
| pet_square_gldm_SmallDependenceLowGrayLevelEmphasis | 0.711 | |  |  |  | |  |
| pet_squareroot_firstorder_Skewness | 0.795 | |  |  |  | |  |
| pet_logarithm_glcm_DifferenceVariance | 0.016 | | 0.017 | 1.001 | 1.000 - 1.002 | |  |
| pet_logarithm_gldm_DependenceVariance | 0.359 | |  |  |  | |  |
| pet_logarithm_ngtdm_Strength | 0.250 | |  |  |  | |  |
| pet_exponential_firstorder_Kurtosis | 0.549 | |  |  |  | |  |
| pet_exponential_glrlm_LongRunHighGrayLevelEmphasis | 0.204 | |  |  |  | |  |
| pet_exponential_glrlm_RunPercentage | 0.054 | |  |  |  | |  |
| pet_exponential_glszm_SmallAreaEmphasis | 0.234 | |  |  |  | |  |
| pet_exponential_glszm_SmallAreaLowGrayLevelEmphasis | 0.075 | |  |  |  | |  |

Table 7 10-fold cross-validation using SVM or LR algorithm for testing the generalization ability of the models

| Parameters/Model | 10-fold cross validation using RF | | | |  | 10-fold cross validation using LR | | | |
| --- | --- | --- | --- | --- | --- | --- | --- | --- | --- |
|  | AUC | Sensitivity (%) | Specificity (%) | Accuracy (%) |  | AUC | Sensitivity (%) | Specificity (%) | Accuracy (%) |
| Combined Model | 0.825 | 78.38% | 76.58% | 77.06% |  | 0.810 | 67.38% | 71.43% | 70.00% |
| PET/CT Radiomic Model | 0.779 | 70.97% | 74.26% | 72.94% |  | 0.740 | 31.64% | 69.56% | 60.00% |
| CT Radiomic Model | 0.745 | 66.38% | 71.37% | 69.41% |  | 0.752 | 46.00% | 65.18% | 62.35% |
| PET Radiomic Model | 0.654 | 51.48% | 69.88% | 63.53% |  | 0.673 | 13.86% | 68.65% | 57.65% |

RF: random forest; LR: logistic regression
