# Supplementary figures and images for: Performance of 18F-FDG PET/CT Radiomics for Predicting EGFR Mutation Status in Patients With Non-Small Cell Lung Cancer
Source: Front Oncol. 2020 Oct 8;10:568857. doi: 10.3389/fonc.2020.568857 (PMC7578399; doi:10.3389/fonc.2020.568857)

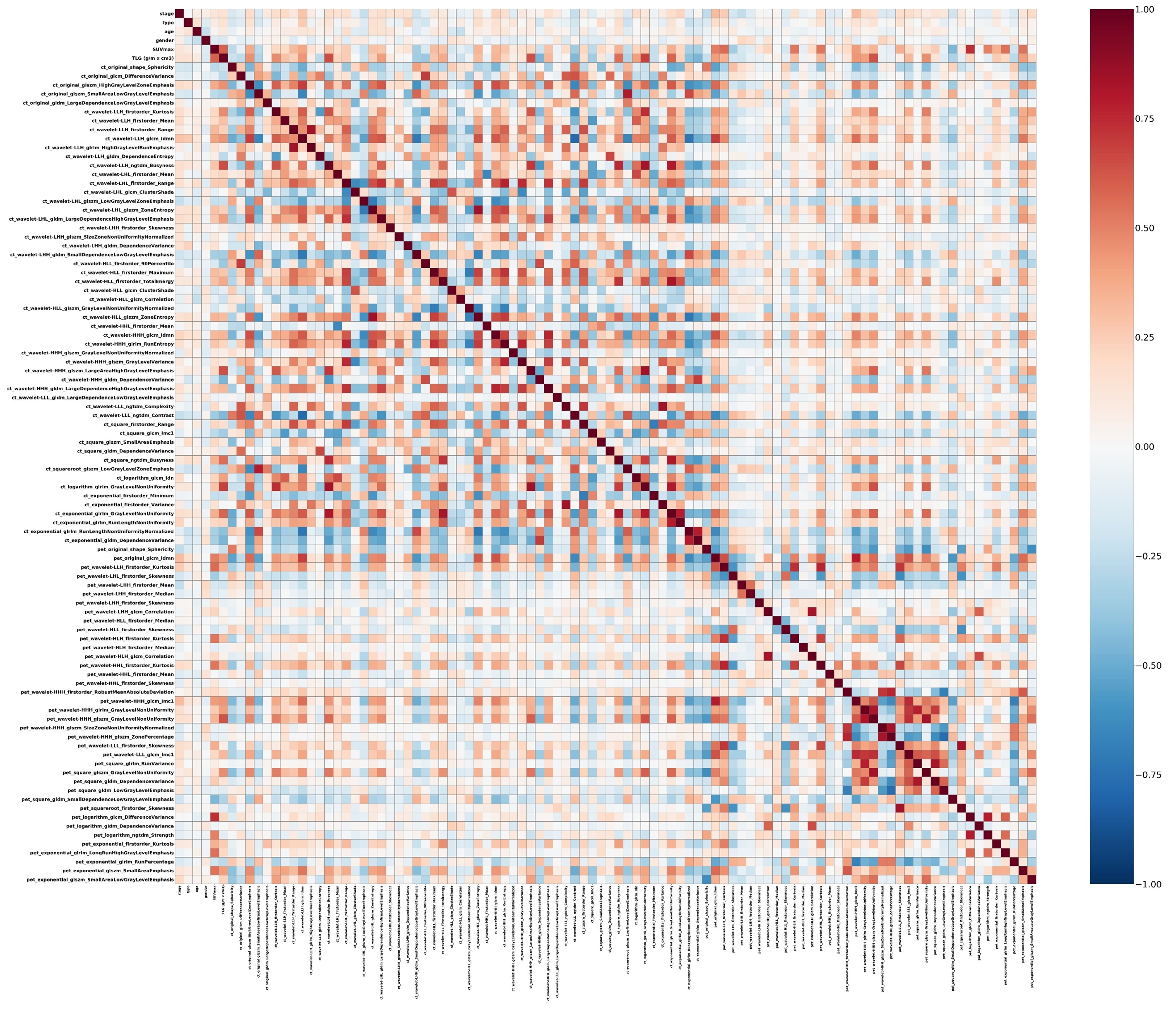

Supplement: Supplementary file 1 [file DataSheet_1.zip › Suppl. Figure 1 Spearman’s correlation coefficient between 100 PETCT radiomic features and 4 conventional PET parameters.jpg]
